# Supplementary material for: The Phenoxyphenol Compound diTFPP Mediates Exogenous C2-Ceramide Metabolism, Inducing Cell Apoptosis Accompanied by ROS Formation and Autophagy in Hepatocellular Carcinoma Cells
Source: Antioxidants (Basel). 2021 Mar 5;10(3):394. doi: 10.3390/antiox10030394 (PMC7998835; doi:10.3390/antiox10030394)
Supplement: Supplementary file 1 [file antioxidants-10-00394-s001.pdf]

antioxidants

Supplementary Materials: The Phenoxyphenol Compound diTFPP Mediates Exogenous C_2-Ceramide Metabolism, Inducing Cell Apoptosis Accompanied by ROS Formation and Autophagy in Hepatocellular Carcinoma Cells

Wen-Tsan Chang [1,2,3†], Yung-Ding Bow [4,†], Yen-Chun Chen [5], Chia-Yang Li [6], Jeff Yi-Fu Chen [5], Yi-Ching Chu [7], Yen-Ni Teng [8], Ruei-Nian Li [9] and Chien-Chih Chiu [3,5,10,11,12,*]

1. Division of General and Digestive Surgery, Department of Surgery, Kaohsiung Medical University Hospital, Kaohsiung 807, Taiwan; wtchang@kmu.edu.tw
2. Department of Surgery, School of Medicine, College of Medicine, Kaohsiung Medical University, Kaohsiung 807, Taiwan
3. Center for Cancer Research, Kaohsiung Medical University Hospital, Kaohsiung Medical University, Kaohsiung 807, Taiwan
4. College of Life Science, Kaohsiung Medical University; Kaohsiung 807, Taiwan; u109850001@kmu.edu.tw
5. Department of Biotechnology, Kaohsiung Medical University, Kaohsiung 807, Taiwan; r020135@gap.kmu.edu.tw (Y.-C.C.); yifuc@kmu.edu.tw (J.Y.-F.C.)
6. Graduate Institute of Medicine, College of Medicine, Kaohsiung Medical University, Kaohsiung 807, Taiwan; chiayangli@kmu.edu.tw
7. Department of Medicinal and Applied Chemistry, Kaohsiung Medical University, Kaohsiung 807, Taiwan; N56094512@ncku.edu.tw
8. Department of Biological Sciences and Technology, National University of Tainan, Tainan 700, Taiwan; tengyenni@mail.nutn.edu.tw
9. Department of Biomedical Science and Environment Biology, Kaohsiung Medical University, Kaohsiung 807, Taiwan; runili@kmu.edu.tw
10. Department of Biological Sciences, National Sun Yat-Sen University, Kaohsiung, 804, Taiwan
11. The Graduate Institute of Medicine, Kaohsiung Medical University, Kaohsiung 807, Taiwan
12. Department of Medical Research, Kaohsiung Medical University Hospital, Kaohsiung 807, Taiwan

* Correspondence: cchiu@kmu.edu.tw; Tel.: +886-7-312-1101 (ext. 2368); Fax: +886-7-312-5339

† The authors contributed equally.

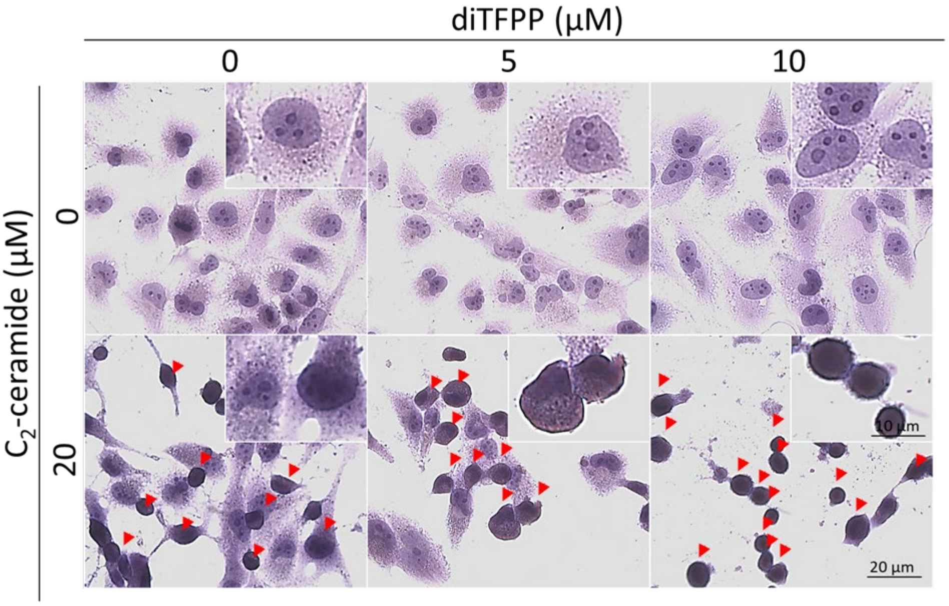

Figure S1. The morphology of HA22T cells with hematoxylin staining after diTFPP/C_2-ceramide treatment. The red arrows indicate pyknotic cells.

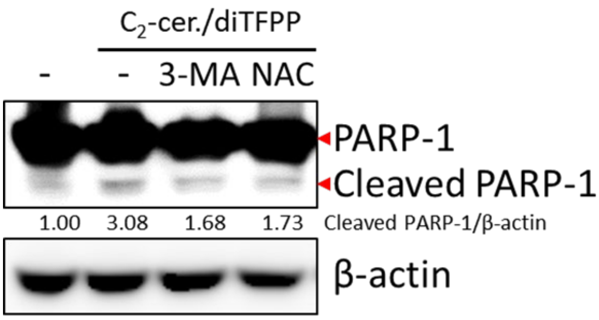

Figure S2. Western blot analysis of PARP-1 expression after C2-ceramide and diTFPP treatment.

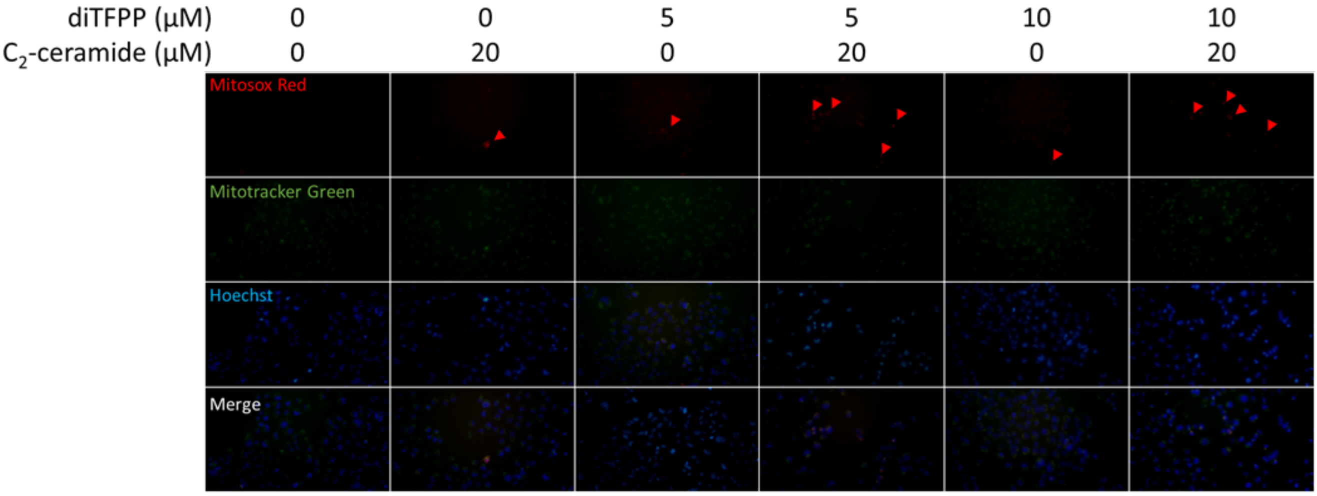

Figure S3. Mitochondrial ROS generation in C2-ceramide- and diTFPP-treated HA22T cells based on MitoSOX Red (red), MitoTracker Green (green), and Hoechst (blue) staining. Red arrows indicate accumulated mitochondrial ROS.
